# Access to advanced healthcare services and its associated factors among patients with cervical cancer in Addis Ababa, Ethiopia

**DOI:** 10.3389/fonc.2024.1342236

**Published:** 2024-02-23

**Authors:** Tariku Shimels, Biruck Gashawbeza, Teferi Gedif Fenta

**Affiliations:** ^1^ Research Directorate, Saint Paul’s Hospital Millennium Medical College, Addis Ababa, Ethiopia; ^2^ Department of Pharmaceutics & Social Pharmacy, School of Pharmacy, College of Health Sciences, Addis Ababa University, Addis Ababa, Ethiopia; ^3^ Department of Gynecology & Obstetrics, Saint Paul’s Hospital Millennium Medical College, Addis Ababa, Ethiopia

**Keywords:** access, Addis Ababa, cervical cancer, Ethiopia, healthcare services, perceived access, realized access

## Abstract

**Objective:**

This study aimed to assess the extent of access to healthcare services, perception and associated factors among patients with cervical cancer in Addis Ababa, Ethiopia.

**Methods:**

A facility-based cross-sectional study was conducted from 01 July through 30 August 2023 at two tertiary hospitals. Cases with histopathologic and clinical confirmation of cervical cancer were enrolled using a consecutive sampling technique. Data was collected through a validated questionnaire administered by interviewers using the KoboCollect application. Subsequently, the collected data underwent analysis using Statistical Sciences for Social Sciences (SPSS) version 26.0. Bivariable and multivariable regression models were performed at p ≤ 0.2 and p<0.05 statistical significance, respectively.

**Results:**

A total of 391 patients were enrolled in the study. Health facilities were accessible for obtaining general medical services (56.5%), drugs (57.3%), laboratory diagnosis services (57.0%), imaging diagnosis services (56.8%), and radiotherapy services (55.8%) of the patients. Cost of services was affordable only in 11.5% of the cases. Essential anticancer medicines were out of stock for 229 (58.6%) of the patients during the past three months. About two-thirds of the patients have a good perception of access to healthcare services. In multivariable binary logistic regression, admission to the inpatient (AOR: 0.20, 95% CI: 0.06-0.67), joblessness (AOR: 0.19, 95% CI: 0.08-0.46), lower level of income to the extreme poverty line (3567ETB)(64.9 USD) (AOR: 0.19, 95% CI: 0.10-0.35), no CBHI coverage (AOR: 4.16, 95% CI: 1.76-9.85), having social support (AOR: 3.80; 95% CI: 1.96-7.41), and poor dietary practice (AOR: 2.36, 95% CI: 1.28-4.35) were found to have a statistically significant association with perceived good access to healthcare services.

**Conclusion:**

Only close to a half of the patients with cervical cancer, in Addis Ababa, have adequate access to healthcare services. Nearly two-thirds of the patients reported perceived good access to the services. Many factors were found to show a statistically significant association with patients’ perceived access to healthcare services.

## 1 Background

Cervical cancer causes a devastating health outcome in women of all age groups, potentially leading to morbidity and premature mortality. Although the disease can be prevented and treated effectively, most notably when detected at an early stage (stages I and II), there is a growing disparity in women’s access to essential healthcare services (1, 2). The problem worsens when more than eight in ten women diagnosed with and nine in ten women who die from cervical cancer live in low-middle-income countries (3). Compared with women in high-income nations, those in low-and middle-income countries have a 35% higher average life risk of cervical cancer globally (4). The average age at diagnosis was reported to be 53 years, ranging from 44 years (Vanuatu) to 68 years (Singapore), and the average age at death from cervical cancer was 59 years, ranging from 45 years (Vanuatu) to 76 years (Martinique) both reported globally (5).

The prevalence of cervical cancer in Ethiopia ranged from 18% to 23% (6, 7). The age standardized incidence and mortality rates in 2020 were about 21.5 and 16.0 per 100000 population respectively (7). Nearly 6,300 new cases are reported to be diagnosed annually, while about 4,884 women die from the same disease each year, making it the second most common and most deadly cancer in this group in the country (8).

Within health service delivery, access is measured in terms of supply of services, affordability, physical accessibility, and acceptability (9, 10). A more comprehensive perspective to encompass the broader aspects of access can also include elements about the structural features of the healthcare system, such as availability; features of individuals, such as preferences, taste, and information (11, 12); and financial, organizational, and professional factors (13). Nonetheless, as healthcare service delivery is not a homogenous product characterized as a single service, the exact meaning of having access remains vague (14). More precisely, it was also depicted as describing the link between specific patient and organizational dimensions (15). This conception labels access in 5As: availability, accessibility, accommodation, affordability, and acceptability. Further to this, supply-side features of health systems, as well as features of populations, along with the processes describing how access is realized, are mentioned as necessary (16, 17).

A multitude of factors may interact with the access of patients to healthcare, including poor cervical cancer screening, lack of awareness and knowledge of cervical cancer, biological factors such as poor nutrition; infections with the human immunodeficiency virus (HIV), tuberculosis (TB), and malaria; and socioeconomic and sociocultural factors with political inequities (18). Further, challenges related to the health system, psychological (fear of recurrence, negative social attitude, and distress associated with the side effects of treatments), and economic factors were identified as the barriers to women’s access to healthcare per a study in Addis Ababa (19). According to recent sources, all such factors could be grouped under three primary types of barriers: structural, financial, and cognitive (20, 21). For example, personal and cultural barriers can inhibit the access or utilization of patients who need medical attention from seeking it or how they act once they obtain the care (22).

To the best of the principal investigator’s knowledge, there is an insufficient understanding of the comprehensive aspects of access to advanced healthcare services for patients with cancer, specifically those with cervical cancer in Addis Ababa. This knowledge gap is noteworthy, especially given the growing incidence of cases and the fact that the city hosts the country’s oldest and referral oncology service settings. Moreover, the perception of patients regarding the current health service delivery and its associated factors has not been determined, despite its considerable potential to impact patients’ utilization of healthcare services and their satisfaction with the care they receive. In other words, the way patients perceive healthcare access is critical, impacting their engagement with the healthcare system, adherence to treatment plans, and overall health outcomes. Through identifying barriers and facilitators at the study settings, these perceptions enable precise interventions and improvements in healthcare delivery.

**Figure 1:**
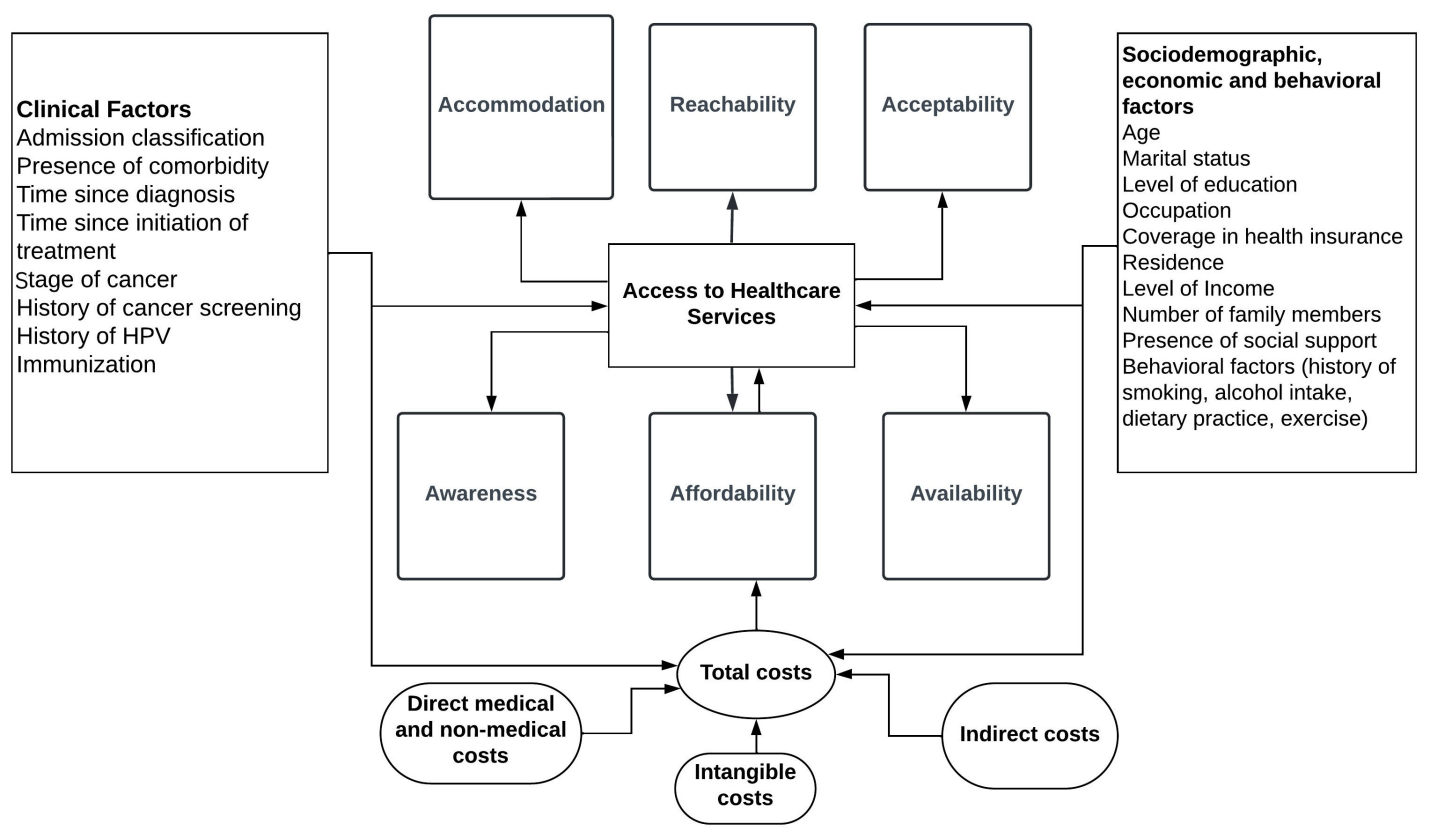
Conceptual model of access to healthcare services among patients with cervical cancer in Addis Ababa, Ethiopia.

In the present study, different social, economic, clinical, and health system-related factors were modeled and assessed against their potential contribution to influencing the extent of access to healthcare of patients with cervical cancer in Addis Ababa, Ethiopia. A conceptual model of this theoretical construct has been depicted in **Figure 1**.

## 2 Methods

### 2.1 Study setting, design, and period

The study was conducted at two leading public oncology centers in Addis Ababa, Saint Paul’s Hospital Millennium Medical College (SPHMMC) and Tikur Anbessa Specialized Hospital (TASH) at Addis Ababa University. The two public hospitals rank as the country’s top referral units, concomitantly delivering all specialized and comprehensive oncology treatment services and medical training in the city. An analytical cross-sectional design was carried out from 01 July through 30 August 2023.

### 2.2 Populations and eligibility criteria

The source population was all women diagnosed with cervical cancer and visiting the selected public and private hospitals in Addis Ababa, Ethiopia. The study population was all patients with a pathologically and clinically confirmed cervical cancer diagnosis who either started or continued a previous treatment in the selected oncology settings during the study period. All adult cases aged 18 or older, residing in Addis Ababa city, started any treatment and were on follow-up at least for a month, could provide information either themselves or through their caregivers, and those who offered verbal informed consent to participate in the study were eligible.

### 2.3 Sample size and sampling method

Taking into consideration the fact that earlier reports were not available on the topic, as well as the intent to increase the required sample size, the single population proportion formula with the following assumptions were used (23). These were i) assuming the probability of perceived adequate healthcare service in 50% of the cases (P=0.5), ii) a 95% level of confidence (α=5%), and iii) a 5% tolerable margin of error. This finally results in a total of at least 385 study participants to be included. As the number of cases visiting the hospitals was limited, a consecutive sampling method was employed. All cases that fulfilled the inclusion criteria and visited the centers during the specified period were assessed.

### 2.4 Data collection instrument and process

Structured psychometric instruments, validated and augmented with a set of objective queries, were utilized for the collection of quantitative data. Both tools were developed as interviewer-administered questionnaires, aiming to comprehensively assess various aspects of access to healthcare services. The design and scope of these instruments were shaped by a thorough literature review. The instrument comprised a total of 68 items, of which nine were on objective measures of access to healthcare services, 19 were on measures of patients’ perception of healthcare services, and the rest were on patients’ socioeconomic, behavioral, and clinical characteristics. The psychometric measures were based on a five-level Likert questionnaire ranging from 1(strongly disagree) to 5 (strongly agree). All questions were designed and loaded in the KoboToolbox open data source server. Exit interviews were conducted using the mobile phone-based KoboCollect or ODK collect application.

### 2.5 Data quality assurance

The following steps were taken into account during the process of this study in order to ensure the quality of data and reports. Firstly, a validated instrument for the psychometric measure was used to ensure that the tool addresses both validity and reliability issues. Further, objective measures were designed and added, translated to Amharic, and then back-translated to English to ensure consistency. Secondly, experienced health professionals, namely three nurses and two general practitioners, were employed for data collection. One-day training on research ethics principles, patient privacy, and data confidentiality was delivered to the data collectors. Thirdly, continuous supervision was maintained across all fieldwork processes to minimize potential errors.

### 2.6 Variables

#### 2.6.1 Dependent variables

The dependent variable in the present study was extent of access to healthcare services, measured using objective also used as ‘realized access’ and subjective methods—the objective methods covered accessibility, affordability, and availability. Accessibility was measured based on the WHO’s definition, in which a health facility was deemed accessible if it is either within 5 kilometers or an hour’s distance from a patient’s home (24). Affordability, on the other hand, was estimated based on the reported current-month patient expenses for treatments in the current illness and income level for the country’s lowest-paid government workers (LPGW). The number of wage days required was computed from the ratio of daily expenses and LPGW rates. A ratio below 1 was assumed affordable and unaffordable otherwise. Availability was calculated based on the observation of data collectors or patient reports on the availability of each service or product during their visit. For palliative care and surgical services, the assumption was whether the facility offered those services or not. The psychometric measure included patients’ perception of healthcare services in terms of five dimensions: acceptability (cultural, social and religious factors of seeking or receiving treatment), availability, accommodability (suitability of the facility’s infrastructure or health-system for patients seeking treatment), affordability (costs assessed against individuals’ ability or willingness to pay), and awareness (patients’ feeling of available treatment options, client-provider interactions and the quality of services provided). The subjective assessment encompassed patients’ perceptions of obtaining follow-up medical care, such as medications, radiotherapy, surgery, consultations, or any combination of these or other treatments to which a patient with a confirmed diagnosis of cervical cancer is entitled. Hence, the scope was limited to those where medical care needs had already been established and did not include those lacking access to cervical cancer screening, precancerous diagnosis, or education services in the community. Perception level was measured on a five-scale Likert questionnaire that ranged between 1 (absolutely disagree) and 5 (absolutely agree), and the aggregate score over the mean was considered ‘‘good access’’ and ‘‘poor access’’ otherwise.

#### 2.6.2 Independent variables

**Socio-demographic and economic characteristics**: these included marital status, literacy status, education level, occupation, religion, average monthly income, residence, number of families, presence of supporting people around, current status of community-based health insurance (CBHI) coverage, and age.

**Clinical characteristics**: these included the presence of comorbidity, the name of comorbidity, time since cervical cancer was first diagnosed, time since treatment began, cancer stage, history of cervical cancer screening, and history of HPV vaccination.

**Lifestyle and substance use-related variables:** included current dietary practice, current physical exercise (adequate vs. inadequate), current history of alcohol intake, current history of smoking cigarettes or using tobacco products, and current history of khat consumption.

### 2.7 Operational definitions

**Physical exercise**: also known as physical activity, was measured based on the recommendations set by the US physical exercise advisory committee (25) and the American Cancer Society (ACS) (26) as well as the World Health Organization (WHO) (27) that patients with chronic illness, particularly those with cancer patients could accommodate engaging in moderate exercise. Accordingly, a patient needs to engage in 150-300 minutes of moderate exercise, or 75-100 minutes of vigorous physical activity per week. Considering these recommendations, a patient was classified to engage in either moderate or vigorous physical activity or not at all, based on the self-reported assessments during the study period.

**Current history of dietary practice**: as defined in the guideline by the American cancer society (26), the dietary recommendation for cancer patients entails taking foods that are high in nutrients in amounts that help them get to and stay at a healthy body weight, a variety of vegetables (dark green, red, and orange, fiber-rich legumes (beans and peas), and others), and fruits, especially whole fruits in a variety of colors, as well as whole grains. In the meantime, it is advised that patients reduce or limit red and processed meats, sugar-sweetened beverages, highly processed foods and refined grain products. This study used these criteria to classify patients’ current dietary practices as “good” (when they report practicing any combination of the recommended diets during the study period) or “poor” (when they report practicing any of the high-risk diets or do not have access to make an adequate diet at all during the study period).

**Current use of excess alcohol**: was defined based on the response of a participant to the question ‘do you currently drink excess alcohol? (i.e >1 bottle a day) that has been set by the American Cancer Society (26).

**Current use of cigarettes**: as per the definition by WHO (27), it refers to the use of cigarettes (smoked or non-smoked) reported by the respondents during the study period.

**Current use of khat or other stimulant substances**: was defined depending on whether a patient reports of using khat or other stimulant substances (i.e Hashish, Ganja or other controlled substances) during the study period.

**Extent of access to healthcare services**: refers to the degree of availability of crucial advanced healthcare services for cervical cancer patients, assessed through either objective or subjective measures as outlined in the ‘dependent variables’ subheading.

### 2.8 Data analysis

Data entered into KoboToolbox was exported to MS Excel, cleaned for completeness, trimmed for unwanted or confidential elements, and coded manually to numerical forms. Quantitative data was then entered into IBM Statistical Packages for Social Sciences (SPSS) for Windows, version 26.0 (IBM Corp., Armonk, N.Y., USA) (28) for further analysis. Apart from presenting univariate and bivariate descriptions of participant attributes, perceived good access to healthcare and associated factors were modeled in binary logistic regression. The bivariate and multivariable logistic regression models were performed at p ≤ 0.2 and p<0.05 levels of significance, respectively, and 95% confidence interval. Model assumption tests, including multicollinearity diagnosis, were performed using variance inflation factor (VIF). While model significance was measured using the Omnibus test, global model goodness of fit was assessed using the Hosmer and Lemeshow test. Similarly, non-parametric tests and chi-squared statistics were used to identify a relationship between realized and perceived access to healthcare services.

### 2.9 Ethical consideration

The study was approved by the institutional review board (IRB) of the School of Pharmacy, College of Health Sciences, Addis Ababa University (Ref.no: ERB/SOP/543/15/2023), and Saint Paul’s Hospital Millennium Medical College (SPHMMC) (Ref.no: PM23/284) before commencing actual data collection. A support letter was written to the selected oncology centers. A verbal informed consent was obtained from every study participant. Participation in the study was voluntary. Data was analyzed in aggregate, and the study did not use personal identifiers. The confidentiality of data collected and the privacy of patients was protected.

## 3 Results

### 3.1 Socioeconomic profile of patients

A total of 391 patients were enrolled in the present study. Six of the extra patients added to the minimum sample size were included following a paper-based completion by one of the enumerators. The mean ( ± SD) age of participants was 50.3(12.2) years ranging from 22 to 89. Similarly, the mean ( ± SD) of participants’ average monthly income was 4960.8(4046.3) Ethiopian birr which ranges from 0 to 50000 ETB. With this, the majority were in the age group of 50 or less (n=238, 60.9%), above the extreme poverty line (n=229, 58.6%), married (n=225, 57.5%), able to read and write (n=243,62.1%), had no formal education (n=166, 42.4%), housewives (n=128, 32.7%), had a community-based health insurance (CBHI) coverage (n=304, 77.7%), had no any form of social support (n=224, 57.3%) and had five or less number of families in the household (328, 83.9%) (**Table 1**).

**Table 1:** Socioeconomic profile of patients with cervical cancer in Addis Ababa, Ethiopia (n=391).

Variable	Label	Frequency	Percentage
Age (Yrs.)	50 or less	238	60.9
	>50	153	39.1
Average monthly income of the household	3567 ETB or less	162	41.4
	>3567 ETB	229	58.6
Marital status	Single	20	5.2
	Married	225	57.5
	Widowed	76	19.4
	Separated or separated	70	17.9
Able to read and write	Yes	243	62.1
	No	148	37.9
Education level	No formal education	166	42.4
	Primary education (grade1-8)	109	27.9
	Secondary education (grades 9-12)	75	19.2
	Tertiary education (University or college)	41	10.5
Occupation	Housewife	128	32.7
	Private employee	121	30.9
	Government employee	29	7.4
	Jobless	74	18.9
	Others*	39	9.9
Community-based health insurance coverage	Yes	304	77.7
	No	87	22.3
Presence of social support	Yes	167	42.7
	No	224	57.3
Household size	5 or less	328	83.9
	>5	63	16.1

*Includes merchants, students, housemaid, farmer.

### 3.2 Behavioral characteristics of patients

**Figure 2:**
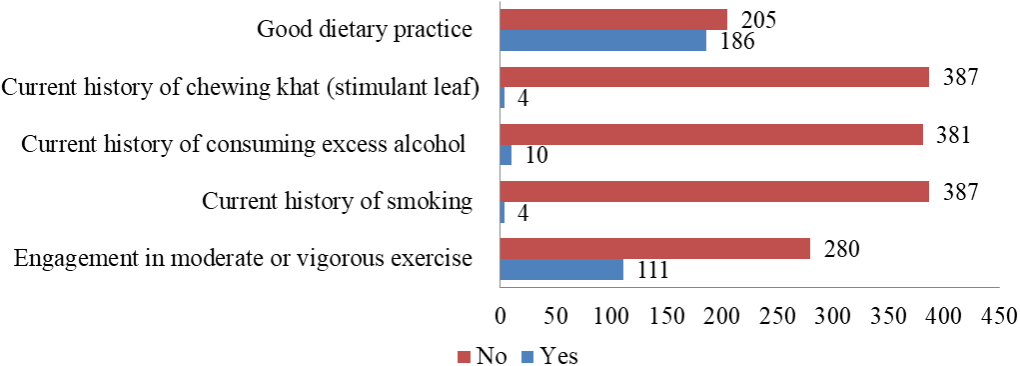
Behavioral characteristics of patients with cervical cancer in Addis Ababa, Ethiopia (n=391).

Assessment of behavioral characteristics of the participants revealed that none were currently utilizing any form of the addictive substance (ganja), whereas only a few consumed cigarettes (n=4), Khat (Khata edulis) (n=4), or an excess level of alcohol (more than one bottle a day) (n=10). Considering dietary habits, the majority (n=205, 52.4%) had poor practices, while the remaining 47.6% had good dietary habits. Meanwhile, over two-thirds of the patients reported that they were currently engaged in either moderate (n=273, 69.8%) or vigorous (n=7, 1.8%) exercise (**Figure 2**).

### 3.3 Clinical profile of patients

Over half (222, 56.8%) of the patients were accessed from Tikur Anbessa Specialized Hospital (TASH). The mean ( ± SD) years since confirmed diagnosis was 1.45(1.25), ranging from 1 month to 10 years, while the mean ( ± SD) of years since any treatment was initiated was 1.36(1.15), which ranges from 1 month to 9 years. Most of the patients were visiting the outpatient department (n=367, 93.9%), nearly half were in stage II of FIGO classification (n=202, 51.7%), three-fourth had no any history of cervical cancer screening (295, 75.4%), nearly one-third received only chemotherapy (n=124, 31.8%) and over a half (n=232, 59.3%) had no any history of chronic comorbidity (**Table 2**).

**Table 2:** Clinical profile of patients with cervical cancer in Addis Ababa, Ethiopia (n=391).

Variable	Label	Frequency	Percent
Hospital	TASH	222	56.8
	SPHMMC	169	43.2
Admission classification	Outpatient	367	93.9
	Inpatient	24	6.1
Stage of disease	Stage I	70	17.9
	Stage II	202	51.7
	Stage III	100	25.6
	Stage IV	19	4.9
History of cervical cancer screening	Yes	96	24.6
	No	295	75.4
Treatments received/being received	Surgery only	45	11.5
	Chemotherapy only	124	31.8
	Radiotherapy only	9	2.3
	Chemotherapy and radiation	24	6.2
	Surgery and radiation	47	12.1
	Chemotherapy and surgery	33	8.4
	Chemotherapy, radiation, and surgery	78	20.0
	Traditional medicine and others*	31	7.9
Presence of any chronic comorbidity	Yes	159	40.7
	No	232	59.3

*includes any combinations of chemotherapy, surgery, or radiotherapy.

### 3.4 Common chronic comorbidities

**Figure 3:**
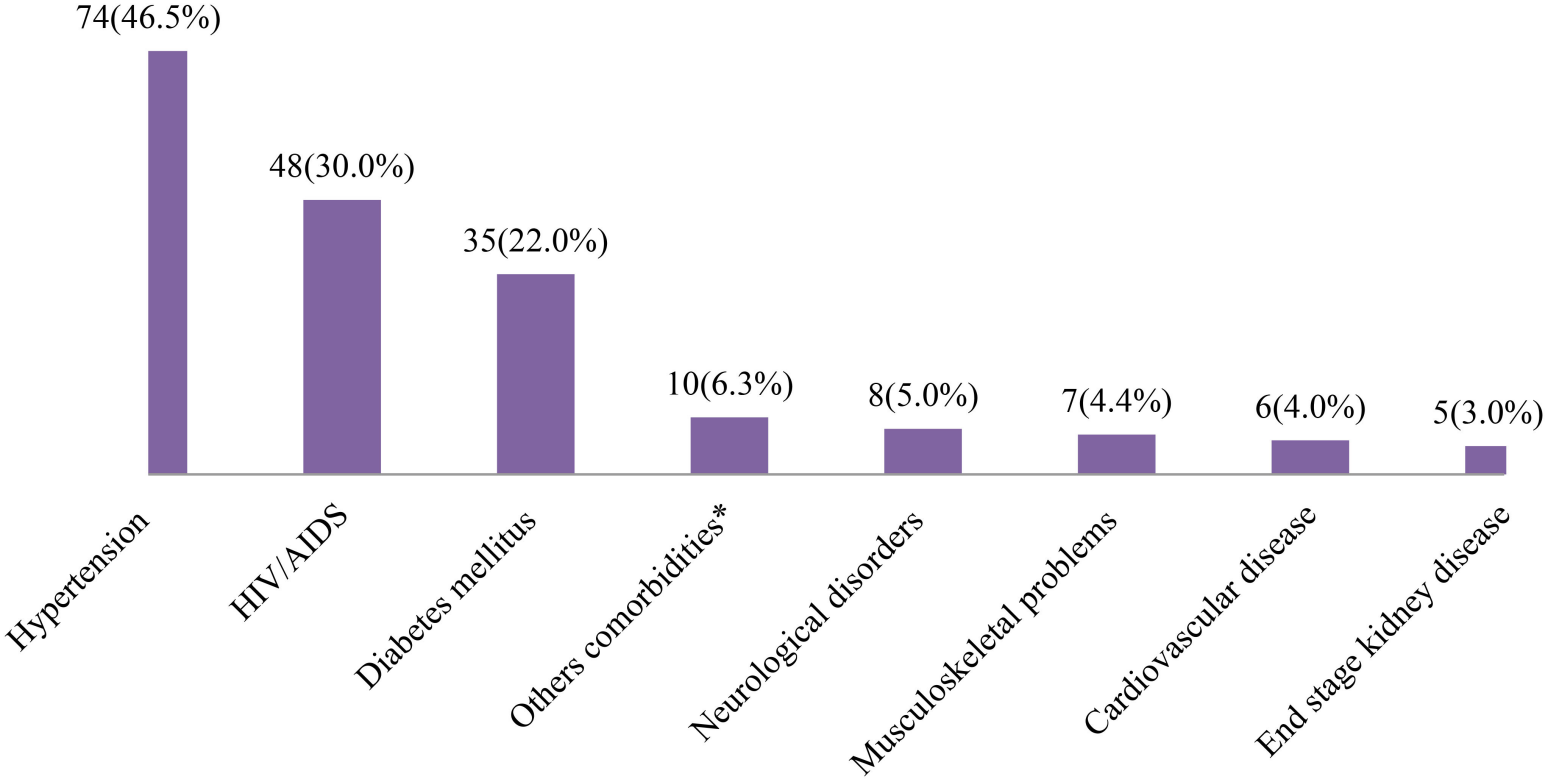
Common comorbidities among patients with patients with cervical cancer in Addis Ababa, Ethiopia (n=159). *eye cataracts, thyroid disease, anemia, lupus or asthma.

Of those who reported having any history of chronic comorbidities, hypertension was reported as the commonest reported by 74(46.5%), followed by human immune deficiency virus/acquired immune deficiency syndrome (HIV/AIDS) (n=48, 30.0%) and type 2 diabetes mellitus (n=35, 22.5%) (**Figure 3**).

### 3.5 Realized access to healthcare services

The realized access to healthcare services was assessed in three dimensions, namely, accessibility, availability, and affordability. The geographic access was measured using the number of minutes it would take for patients to reach the nearest health facilities to obtain different healthcare services related to their current illness. Accordingly, health facilities are accessible in obtaining general medical services in 221 (56.5%), drugs in 224 (57.3%), laboratory diagnosis services in 223 (57.0%), imaging diagnosis services in 222 (56.8%), and radiotherapy services in 218 (55.8%) patients. Among the patients with no CBHI coverage (n=87), the health services offered in the facilities were affordable only for 10 (11.5%). On the other hand, evaluating the availability of any of the selected essential anticancer medicines during the past three or fewer months revealed that most were out of stock for 229 (58.6%) of the patients at least once. On the contrary, any basic diagnostic services required for the current illness were available most of the time (n=372, 95.1%) (**Table 3**). In this line, surgical and palliative care services were also available at the health facilities.

**Table 3:** Objective measures of access to healthcare services among patients with cervical cancer in Addis Ababa, Ethiopia (n=391).

Variable	Category	Frequency	Percent
Nearest health facility from home to seek general medical consultation services for the current health problem	Accessible	221	56.5
	Inaccessible	170	43.5
Nearest health facility from home to get drugs for the current health problem	Accessible	224	57.3
	Inaccessible	167	42.7
Nearest health facility from home to get laboratory diagnosis services for the current health problem	Accessible	223	57.0
	Inaccessible	168	43.0
Nearest health facility from home to get imaging diagnostic services for the current health problem	Accessible	222	56.8
	Inaccessible	169	43.2
Nearest health facility from home to get radiotherapy for the current health problem	Accessible	218	55.8
	Inaccessible	173	44.2
Affordability of cost of healthcare services received (n=87)*	Affordable (<1 day’s wage)	10	11.5
	Unaffordable	77	88.5
Availability of any of the essential generic drugs (cisplatin, carboplatin, bleomycin, paclitaxel/docetaxel, rituximab, bevacizumab, gemcitabine) or their brand counterparts at the health facility during the study period (n=259)	Yes	107	41.4
	No	252	58.6
Availability of basic diagnostic services (laboratory, ultrasound, histopathology) required for the current illness	Yes	372	95.1
	No	19	4.9

*Estimation made for non-insured patients and an LPGW daily wage of 50 ETB per minimum wage reported in 2015 (29) and method used elsewhere (30).

### 3.6 Perceived access to healthcare and factors associated with perceived good access to healthcare services

In two-thirds of the patients assessed (n=266, 68.0%), the perceived access to healthcare services was rated good. A binary logistic regression was performed between the dependent variables and independent variables. Variable which fulfilled the bivariable analysis (p≤0.2), namely service classification, ability to read and write, occupation status, household health insurance coverage, average monthly income of the household, the household size, presence of social support, dietary practice, age category, and lifestyle were included in the multivariable model.

The Omnibus test coefficient revealed that the final (fit) model was statistically significant compared to the null model with χ^2^(16) = 194.47, p< 0.001. Global model fitness test of Hosmer and Lemeshow statistics showed a good fit with X^2^(8)=10.40, p=0.24. The model explained 55.0% (NagelkerkeR^2^) of the variance in perceived access to healthcare and correctly classified 85.7% of cases. There was no multicollinearity noted between the independent variables (VIFs ranged between 1.1 and 1.4). Neither was there an interaction identified between suspected factors and covariates.

The multivariable binary logistic regression analysis demonstrated that the perceived good access to healthcare services among inpatients was about 80% lower (AOR: 0.20, 95% CI: 0.06-0.67) than the outpatients. Similarly, as compared with housewives, patients who had no job and others, including merchants, farmers, students as well as housemaids, were about 81% (AOR: 0.19, 95% CI: 0.08-0.46) and 70% (AOR: 0.30, 95% CI: 0.11-0.84) less likely to have perceived good access to healthcare services, respectively. Finally, in this category, patients with lower levels of income to the extreme poverty line (3567ETB) (64.9USD) were 81% (AOR: 0.19, 95% CI: 0.10-0.35) less likely to have perceived good access to healthcare services.

On the other hand, patients with no CBHI coverage were 4.2 times more likely to have perceived good access (AOR: 4.16, 95% CI: 1.76-9.85) than those with CBHI coverage. Patients who reported to have any form of social support were 3.81 times (AOR: 3.81; 95% CI: 1.96-7.41) more likely to have perceived good access than those without social support. Finally, patients with poor dietary practice were 2.36 times (AOR: 2.36, 95% CI: 1.28-4.35) more likely to have perceived good access to healthcare services (**Table 4**).

**Table 4:** Factors associated with perceived good access to healthcare services among patients with cervical cancer in Addis Ababa, Ethiopia (n=391).

Variable	Category	Perceived access to healthcare services		COR	95%CI for COR		AOR	95% CI for AOR	
		Poor (n)	Good (n)		Lower	Upper		Lower	Upper
Service classification	Outpatient	103	264	Ref			Ref		
	Inpatient	19	5	0.10	0.04	0.28	0.20*	0.06	0.67
Marital status	Single	4	16	Ref			Ref		
	Married	64	161	0.63	0.20	1.95	1.35	0.28	6.58
	Widowed	24	52	0.54	0.16	1.79	3.40	0.60	19.25
	Separated or divorced	30	40	0.33	0.10	1.10	1.33	0.25	7.14
Able to read and write	Yes	56	187	Ref			Ref		
	No	66	82	0.37	0.24	0.58	0.93	0.46	1.86
Occupation	Housewife	29	99	Ref			Ref		
	Private employee	25	96	1.13	0.62	2.06	0.50	0.20	1.27
	Government employee	4	25	1.83	0.59	5.69	0.66	0.15	2.89
	Jobless	46	28	0.18	0.10	0.33	0.19*	0.08	0.46
	Others**	18	21	0.34	0.16	0.73	0.30*	0.11	0.84
CBHI coverage***	Yes	111	193	Ref			Ref		
	No	11	76	3.97	2.03	7.80	4.16*	1.76	9.85
Household’s average monthly income	≤3567 ETB	92	70	0.12	0.07	0.19	0.19*	0.10	0.35
	>3567 ETB	30	199	Ref			Ref		
Number of family members	Five or less	96	232	1.70	0.98	2.96	1.43	0.66	3.07
	>5	26	37	Ref			Ref		
Presence of social support	Yes	33	134	2.68	1.68	4.26	3.81*	1.96	7.41
	No	89	135	Ref			Ref		
Dietary habit	Poor	41	164	3.09	1.97	4.83	2.36*	1.28	4.35
	Good	81	105	Ref			Ref		
Age category (Yrs.)	50 or less	52	186	3.02	1.94	4.70	1.91	0.89	4.14
	>50	70	83	Ref			Ref		
Physical exercise	Poor	76	197	1.66	1.05	2.61	1.01	0.52	1.94
	Good	46	72	Ref			Ref		

*Indicates statistically significant association; **Includes merchants, students, housemaids, and farmers; ***Community-based health insurance; COR, Crude Odds Ratio; AOR, Adjusted Odds Ratio.

Model fitness results: Omnibus test of fit model: X^2^(16)=194.67; p<0.001; Hosmer & Lemeshow test:X^2^(8)=10.40; p=0.24; Nagelkerke R^2^:55.0%; Test of multicollinearity: VIF of 1.1 to 1.4.

## 4 Discussion

This study attempted to assess the extent of access to healthcare that patients with cervical cancer have had and the associated factors within an urban context in Ethiopia. Utilized were both actual and perceived measures of access, which, in turn, were guided by a theoretically grounded conceptual model. Even though the cancer care continuum is complex, encompassing all services from screening to palliative care, this study focused on interim cancer care when patients demand accessibility to treatment options after a confirmed diagnosis.

The measure on realized availability of the essential services uncovered that slightly over half (range: 55%–57%) of the patients had access to such healthcare services as medical consultation, drugs, laboratory diagnosis, imaging, and radiotherapy. This suggests that many patients still lacked access to essential healthcare services in the city. There was a concordance between the reports of those with a relatively higher level of realized access and those who perceived good access to healthcare services (**Supplementary Material**). A recent qualitative study that engaged senior providers in one of the study settings remarked that the lack of an adequate number of oncologists, oncology nurses, radiotherapy technologists, and medical physicists was a challenge while the number of cancer cases was growing (31). The obtained figure on accessibility of most of the services, in the current study, is much lower than a report from cancer survivors in the United States, which outlined that only 6% of the patients had reported not to receiving general medical care services required in this group of patients (32).

Evaluating the cost of healthcare services received at the health facilities, it was found that only a small proportion (11.5%) of the patients could afford them, indicating that the services were unaffordable for nearly the entire population. The affordability of the services in the present settings was still lower compared to the figure reported in the latest study conducted in Rwanda, where 20% of the anticancer medicines were affordable (33). One potential difference between the results could be that the current study considered all estimated patient expenses for services received in the month, while the latter evaluated only medicines. We considered the wage days to calculate affordability; the method used in the latter was not clearly stated.

The availability of essential diagnostic services in the study settings was reported to be maintained during most of the time patients visited the facilities (95%), while essential anticancer drugs were available at a lower percentage (41%). The figure is the same as the one reported at public hospitals in Rwanda (33). A study in Mexico reported that the availability of anticancer drugs at public hospitals was 61.2% (34).Though the difference in the latter could be assumed to happen as a result of a better health system in a middle-income country, the finding revealed that the availability of anticancer medicines was far behind the recommended 80% target of the WHO (35).

Nearly two-thirds of the patients with cervical cancer in Addis Ababa have perceived good access to healthcare services. The low perception in a third of the cases could reflect the lack of sufficiency in the physical, financial, and system-level factors that patients face across the continuum of medical care, which, in turn, is close to the figure in the realized access. The report by Haileselassie et al. (31)demonstrated that the perception of patients with cancer about themselves and their trust in the health system remains challenging, along with external factors such as providers, administration, and technology. According to Bourque and Loiselle (2022) (36), how healthcare services are structured, including setting, schedule, and location, are critical in cancer treatment that patients would raise concerns about and influence their perception. From the findings, one could note that realized poor experience during the uptake of a health service can prompt patients to perceive it negatively, influencing their poor utilization of health services provided. One study highlighted that the lack of high-technology equipment, poor coordination, and unskilled professionals would be barriers to quality oncology services (37).

Assessment of factors associated with perceived good access to healthcare services revealed that inpatients, average monthly income below the extreme poverty line, occupation groups, namely jobless and others (merchants, students, farmers and housemaids) were less likely to have perceived good access to healthcare. The lower rate of perception of adequate access among inpatients could be derived from the fact that hospitalization is associated with high costs and the availability of adequate health infrastructure compared with ambulatory services. On the contrary, the hospitals lack sufficient resources. Recent studies indicated that lack of supplies, trained professionals, and service costs were among the common challenges to delivering quality healthcare services at Tikur Anbessa Specialized Hospital (TASH) (31, 38). Patients’ income level, either independently (jobless) or indirectly through the aggregate variable ‘others ‘, can also be a significant driver of perceived poor access to healthcare services in the current setting. This is likely because, patients with a relatively higher income level can afford direct and indirect medical or nonmedical expenses compared to those with lower monthly earnings or no job. Generally, lower income is associated with negative utilization of cancer care (39). On a further diagnosis, it was noted that there was a significant difference in average monthly income level of the households between housewives and those reporting as ‘jobless’ (mean difference: 2267.8; 95% C.I: 678.7-3856.8, p=0.001). On the other hand, the group coded as ‘others’ was comprised of 79.5% identified as merchants among whom 71% were members of the CBHI scheme. A possible account on the inverse relationship between CBHI coverage and perceived good access to healthcare has been elucidated in the next paragraph.

On the contrary, patients with no CBHI coverage, those with poor dietary habits, and those with good social support were more likely to have perceived good access to healthcare services. The observed negative relationship between CBHI scheme coverage and good perceived access to healthcare might not be direct, which, in turn, is against the usual understanding. Acting as an intermediary, the extent of patient satisfaction could predominantly affect patients’ perceptions of quality in healthcare services rendered (40). Patients enrolled in a CBHI scheme have been reported to have poor or moderate satisfaction with the services they received (41). This, in turn, could be attributed to patients’ increased demand and expectation to consume quality health services following their investment in the scheme compared to those not enrolled in it. This also has a link with the perceived acceptability, accommodation and awareness domains of access. On a related note, the positive association between poor dietary habits and perceived good access to healthcare services might be indirect, as the observed relationship between the two variables appears to be quite unapparent. From the chi-square analysis, it was noted that a higher proportion of patients with poor dietary habits were on outpatient visits (54.0%) as compared with those attending the centers as inpatients (29.2%) (p=0.021). Social support is integral to cancer care as it combines and optimizes patient factors, psychosocial and economic issues, and health system-related dimensions. A recent study in one of the current settings revealed that family caregivers had an immense role in patients’ treatment uptake, enhancing patient-provider communication and providing psychosocial support (42).

This study has employed both objective and subjective measures of access to assess the current extent of health service accessibility for patients with cervical cancer. Strong adherence to statistical model assumptions has also been followed to ensure the validity of the findings to be generalized across Ethiopian settings. Efforts were made to minimize potential bias introduced during data collection. With this, assessing access to healthcare services among patients with cervical cancer in Addis Ababa provides a comprehensive understanding of the challenges they face and informs targeted interventions to improve healthcare delivery, reduce disparities, and enhance overall health outcomes. Nonetheless, there were limitations to this assessment. Firstly, the study was limited only to the population in Addis Ababa City, and the findings generated may not reflect the actual extent of the situation in other contexts of the country. Secondly, the scope of healthcare services considered in the work entailed mainly treating confirmed cases; hence, it did not embrace all packages of essential health services, such as screening and diagnosis.

## 5 Conclusion

The present study revealed that nearly half of the patients with cervical cancer in Addis Ababa have optimal access to most of the healthcare services required for their treatment. Lower income levels to the extreme poverty line, occupation groups with no job or farmers, and admission to the inpatient were negatively associated with perceived good access to healthcare services. In contrast, absence of CBHI coverage, social support, and poor dietary adherence were positively associated with perceived good access to healthcare services. The Ministry of Health should take steps to map and facilitate the expansion of cancer care centers equipped with complete infrastructure to low-access areas. Specifically, health leaders should map drug availability, allocate resources strategically, strengthen the supply chain, explore partnerships, enforce regulations, invest in capacity building, establish monitoring systems, conduct patient education, collaborate internationally, and offer incentives to the pharmaceutical industry. Efforts by health facilities to improve the quality of services offered irrespective of CBHI coverage status could impact patients’ satisfaction positively. Existing facilities should equip themselves regarding the availability of drugs, health technologies, and trained providers. Finally, patient education and public awareness addressing illiteracy related to cancer prevention, detection, and management should be actively implemented across health facilities and schools. Future research should also involve patients with other types of cancer and employ various methodologies, such as concentration indicators and the Gini coefficient, to evaluate healthcare access comprehensively across all regions in the country.

## Data availability statement

The original contributions presented in the study are included in the article/**Supplementary Material**. Further inquiries can be directed to the corresponding author.

## Ethics statement

The studies involving humans were approved by Saint Paul Hospital Millennium Medical College IRB and Addis Ababa University, School of Pharmacy IRB. The studies were conducted in accordance with the local legislation and institutional requirements. The ethics committee/institutional review board waived the requirement of written informed consent for participation from the participants or the participants’ legal guardians/next of kin because of no greater than minimal risk due to the research.

## Author contributions

TS: Conceptualization, Data curation, Formal analysis, Funding acquisition, Investigation, Methodology, Project administration, Supervision, Validation, Writing – original draft, Writing – review & editing. BG: Supervision, Validation, Writing – review & editing. TG: Conceptualization, Methodology, Supervision, Validation, Writing – review & editing.
