# Supplementary material for: Access to advanced healthcare services and its associated factors among patients with cervical cancer in Addis Ababa, Ethiopia
Source: Front Oncol. 2024 Feb 23;14:1342236. doi: 10.3389/fonc.2024.1342236 (PMC10921226; doi:10.3389/fonc.2024.1342236)
Supplement: Supplementary file 1 [file DataSheet_1.pdf]

Figures

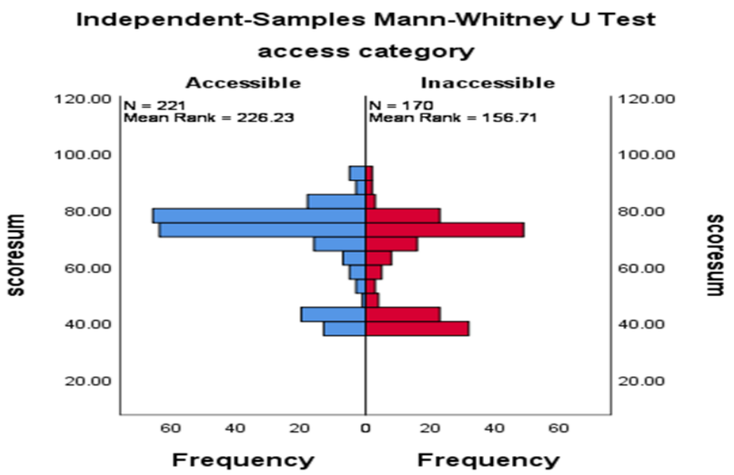

Supplementary figure1: Accessibility of general medical services vs. perceived access to healthcare

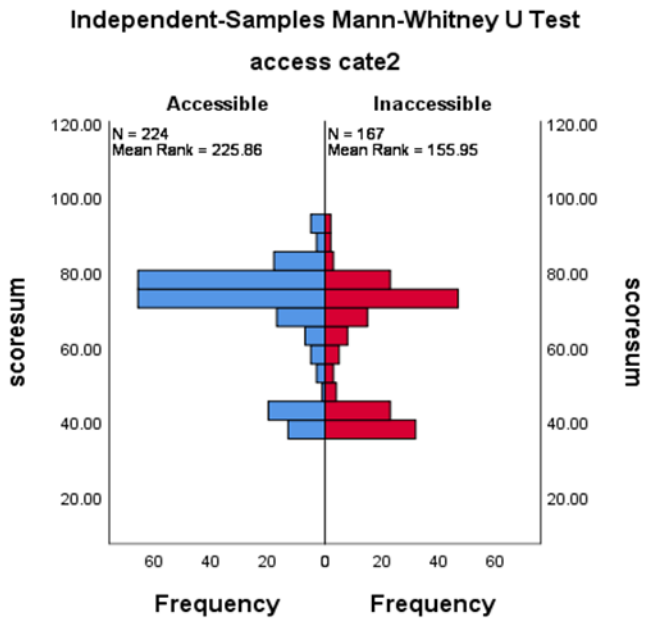

Supplementary figure 2: Accessibility of drugs vs. perceived access to healthcare

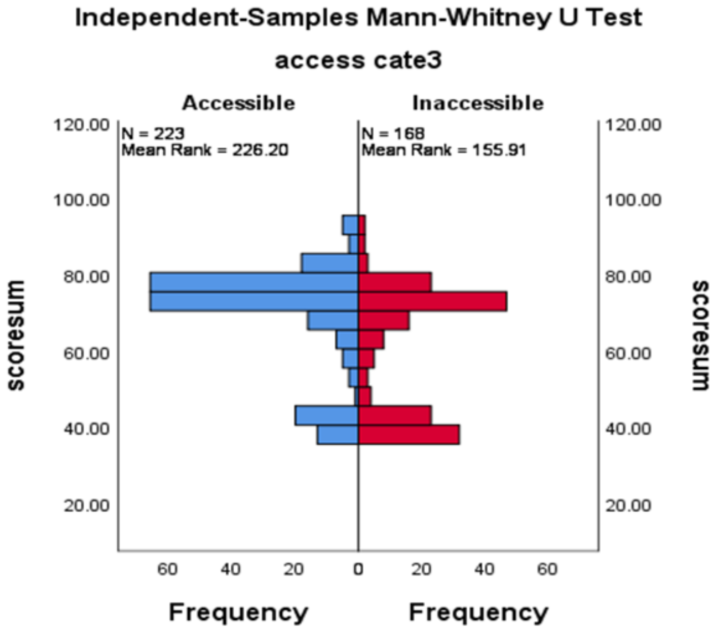

Supplementary figure 3: Accessibility of laboratory services vs. perceived access to healthcare

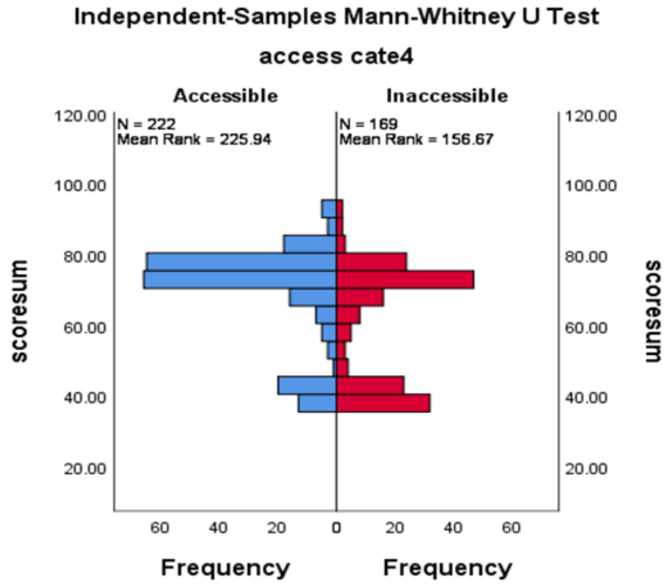

Supplementary figure 4: Accessibility of imaging services vs. perceived access to healthcare

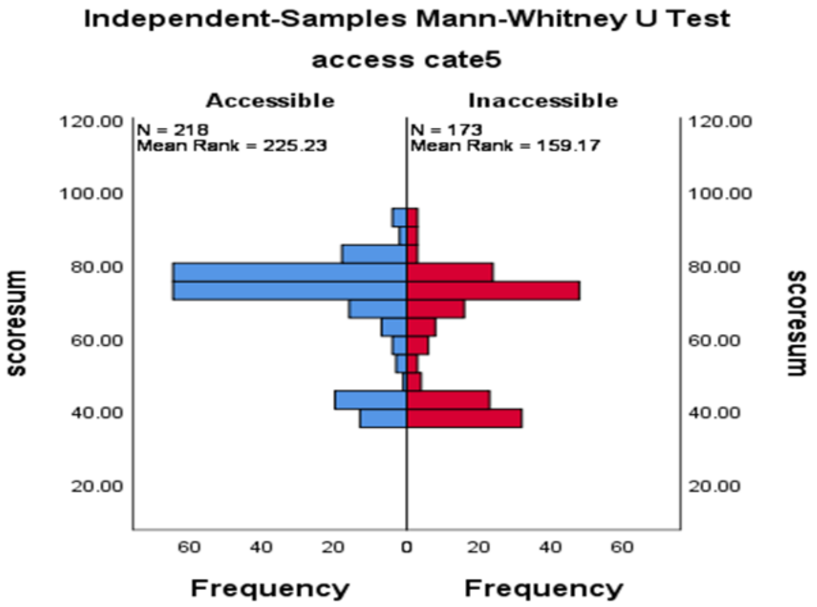

Supplementary figure 5: Accessibility of radiotherapy services vs. perceived access to healthcare
